# Effect of anthelmintic treatment on leptin, adiponectin and leptin to adiponectin ratio: a randomized-controlled trial

**DOI:** 10.1038/nutd.2017.37

**Published:** 2017-10-16

**Authors:** D L Tahapary, K de Ruiter, I Martin, E A T Brienen, L van Lieshout, Y Djuardi, C C Djimandjaja, J J Houwing-Duistermaat, P Soewondo, E Sartono, T Supali, J W A Smit, M Yazdanbakhsh

**Affiliations:** 1 Department of Internal Medicine, Division of Endocrinology, Faculty of Medicine Universitas Indonesia, Dr Cipto Mangunkusumo National General Hospital, Jakarta, Indonesia; 2 Department of Parasitology, Leiden University Medical Center, Leiden, The Netherlands; 3 Nangapanda Community Research Cluster, The Indonesian Medical Education and Research Institute, Universitas Indonesia, Jakarta, Indonesia; 4 Metabolic, Cardiovascular, and Aging Research Cluster, The Indonesian Medical Education and Research Institute, Universitas Indonesia, Jakarta, Indonesia; 5 Department of Medical Statistics and Bioinformatics, Leiden University Medical Center, Leiden, The Netherlands; 6 Department of Mathematics, Parahyangan Catholic University, Bandung, Indonesia; 7 Department of Parasitology, Faculty of Medicine Universitas Indonesia, Jakarta, Indonesia; 8 Department of Statistics, University of Leeds, Leeds, UK; 9 Department of Internal Medicine, Radboud University Medical Centre, Nijmegen, The Netherlands; 10 Department of Internal Medicine, Leiden University Medical Center, Leiden, The Netherlands

## Abstract

Emerging evidence suggests that helminths might confer protection against the development of type 2 diabetes. We aimed to assess the role of adipokines in mediating the effect of helminths on insulin resistance. Serum samples were obtained from a randomized-controlled trial of anthelmintic treatment in an area endemic for soil-transmitted helminths (STH), Flores Island, Indonesia. In STH-infected subjects, anthelmintic treatment significantly increased the ratio of leptin to adiponectin (treatment effect factor (95% confidence interval (CI)), *P*-value for interaction: 1.20 (1.06–1.35), *P*=0.010), which largely stemmed from a significant reduction in adiponectin (0.91 (0.85–0.98), *P*=0.020) and a trend for an increase in leptin level (1.10 (1.00–1.21), *P*=0.119). No significant effect on resistin level was observed. This increase in leptin to adiponectin ratio seemed to contribute to the observed effect of deworming on increased insulin resistance (IR) as adjustment for leptin to adiponectin ratio attenuated the effect on IR from 1.07 (1.01–1.14, *P*=0.023) to 1.05 (0.99–1.11, *P*=0.075). Anthelmintic treatment in STH-infected subjects increases leptin to adiponectin ratio which may in small part contribute to the modest increase in IR. Further studies will be needed to assess the effect of the changes in adipokine levels on the host immune response and metabolism.

## Introduction

Emerging evidence suggests that helminths might confer protection against the development of type 2 diabetes (T2D),^1, 2, 3, 4, 5^ presumably by modulating the host immune responses.^6, 7, 8^ Thus, in addition to the more established risk factors, such as sedentary lifestyle and high-energy foods, current deworming programs in parallel with rapid socioeconomic development might potentially contribute to the development of T2D in many low and middle-income countries.^6^ In line with this, we have recently reported that removal of helminth infections increases insulin resistance (IR),^9^ which is mainly mediated by the increase in adiposity,^9^ suggesting a central role of adipose tissue (AT).^10, 11, 12, 13^

Human AT secretes various adipokines, most notably leptin and adiponectin, affecting metabolic homeostasis and immune regulation.^14^ Leptin and adiponectin have been consistently shown to be positively and negatively associated with IR, respectively.^14^ Whereas leptin promotes pro-inflammatory immune responses and inhibits the proliferation of regulatory T cells, adiponectin induces the secretion of anti-inflammatory cytokines.^15^ The imbalance between those two adipokines, leptin to adiponectin (L/A) ratio, has been reported to be associated with pro-inflammatory conditions and IR.^16, 17^

Assessment of adipokines might provide a valuable insight into the role of human AT in mediating the helminths effect on metabolic homeostasis. To our knowledge, no studies have been published so far on the association between helminth infections and adipokines, except for resistin.^18^ Therefore, we measured leptin, adiponectin, and resistin in serum samples obtained from a randomized-controlled trial of anthelmintic treatment in an area endemic for soil-transmitted helminth (STH).^19^ We hypothesized that the increase in IR after anthelmintic treatment in helminth-infected subjects might be mediated by a shift in L/A ratio towards a more pro-inflammatory state.

## Materials and methods

This present study is part of a household-based cluster-randomized double-blind placebo-controlled anthelmintic trial (The Sugarspin study), conducted in Nangapanda, Flores, an endemic area for STH.^19^ The primary outcome of the Sugarspin study is changes in IR, as assessed using the homeostatic model assessment of IR (HOMA-IR), after anthelmintic treatment, which has been published recently.^9^ Written informed consent was obtained from all participants. The study was approved by the ethics committee of Faculty of Medicine, Universitas Indonesia (FKUI) (ref: 549/H2·F1/ETIK/2013), and filed by the ethics committee of Leiden University Medical Center (LUMC). The trial is registered as a clinical trial (http://www.isrctn.com/ISRCTN75636394).

The population was randomized at household level. After randomization, all subjects in the study area, except children <2 years old and pregnant women, received a single tablet of albendazole (400 mg) or matching placebo for three consecutive days with direct supervision. This three-monthly treatment regimen was given for four rounds. All subjects ⩾16 years old were invited to undergo clinical measurements and blood drawing after an overnight fast, at baseline and 6 weeks after the end of the fourth treatment round (follow-up).^19^ All subjects without sufficient sera samples, and/or incomplete data on body mass index and STH infection status at baseline were excluded from the present study. Subjects receiving active treatment for diabetes were also excluded from analysis.

Body weight and height were measured, and body mass index was calculated as weight (kg) divided by square of height (m). Adipokines (leptin, adiponectin and resistin) were measured by ELISA using commercial reagents (DuoSet ELISA R&D System Europe Ltd, Abingdon, UK), according to the manufacturer’s protocol. Leptin to adiponectin (L/A) ratio was calculated by L/A=leptin level (ng ml^−1^)/adiponectin level (μg ml^−1^).^17^ Soil-transmitted helminth infection status was assessed using both microscopy (Kato Katz) and PCR, which was further stratified by the number of species a subject was infected with at baseline (no infection, single infection, multiple infection).^9^

### Statistical analysis

Leptin, adiponectin, L/A ratio and resistin were log-transformed (log10) for analysis and summarized as geometric mean (95% confidence interval (CI)). The effect of anthelmintic treatment on adipokine was assessed using mixed models to account for the correlation within households, as described previously.^9^ The treatment effect estimates were the regression coefficient obtained from mixed models (β) indicating changes in log10 (leptin, adiponectin, L/A ratio, resistin) of subjects using albendazole compared with placebo. The treatment effect factors (10^β^) are multiplicative instead of additive. Thus treatment effect factors indicate the proportional change for each variable (leptin, adiponectin, resistin, L/A ratio), in comparison to the placebo. All models were fitted using the lme4 package (R software x64 version 3.2.2 for Windows, R Foundation for Statistical Computing, Vienna, Austria, www.r-project.org).

## Results

At baseline, the prevalence of STH infection was 42.0% (503/1195) and 54.1% (760/1405), as assessed by microscopy and PCR, respectively. Serum leptin, adiponectin, L/A ratio and resistin levels were similar in both treatment arms (Table 1). The consort diagram of the present study is shown in Supplementary Figure 1.

**Figure 1:**
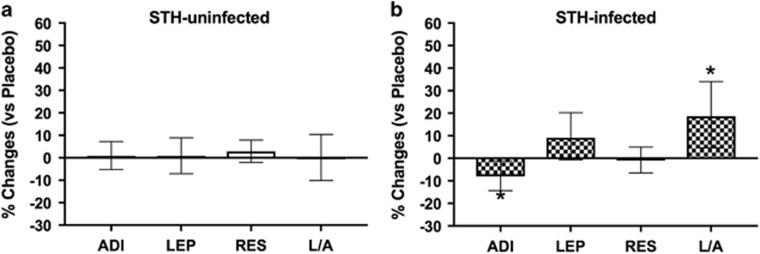
Effect of anthelmintic treatment on adiponectin, leptin, resistin, and leptin to adiponectin ratio in soil-transmitted helminth (STH)-infected and uninfected subjects. The effect of anthelmintic treatment on adiponectin (ADI), leptin (LEP), resistin (RES) and leptin to adiponectin ratio (L/A) in (**a**) STH-uninfected and (**b**) STH-infected subjects, as assessed by microscopy, are presented as proportion of changes (95% CI) between pre and post treatment in the albendazole group compared with the placebo group which is set to zero. Adiponectin, leptin, resistin and L/A ratio were log-transformed for analysis. Analysis was performed on 1183 subjects, after excluding 12 subjects with diabetes. Treatment effect estimates were the regression coefficient (β) obtained from mixed models indicating changes in log (ADI or LEP or RES or L/A); the treatment effect factors (10^β^) are proportional instead of additive. Thus, treatment effect factors indicate the proportional change in each variable in comparison to the placebo group. *P<0.05.

Similar to the main study, anthelmintic treatment significantly reduced the prevalence of STH infections, as assessed by microscopy or PCR (Supplementary Table 1). In comparison to placebo, albendazole treatment had no effect on adipokine levels in subjects without STH infections (Figure 1a). In STH-infected subjects, as assessed by microscopy, albendazole treatment increased L/A ratio (treatment effect factor (95% CI): 1.20 (1.06–1.35), *P*-value for interaction=0.010), which was mostly derived from a significant reduction in adiponectin level (0.91 (0.85–0.98), *P*=0.020) and a trend for an increase in leptin level (1.10 (1.00–1.21), *P*=0.119) (Figure 1b). No significant treatment effect on resistin level was observed (1.00 (0.94–1.05), *P*=0.363; Figure 1b)).

Pathway analysis showed that adjustment for changes in body mass index partly attenuated the treatment effect on L/A ratio (from 1.20 (1.06–1.35) to 1.13 (1.02 – 1.26), *P*=0.040), which translates from 20 to 13%. We also assessed whether the increase in L/A ratio contributes to the increased IR after treatment in helminth-infected subjects.^9^ This analysis showed that adjustment for changes in L/A ratio, attenuated the treatment effect on IR from 1.07 (1.01–1.14, *P*=0.023) to 1.05 (0.99–1.11, *P*=0.075), even more than adjustment for changes in body mass index (Supplementary Table 2).

When light infections were also considered by using PCR, albendazole treatment did not significantly increase L/A ratio (1.10 (1.00 –1.22), *P*=0.321), despite a significantly reduced adiponectin level (0.94 (0.88–0.99), *P*=0.060). No significant treatment effect was observed on the level of leptin, nor resistin (Supplementary Figure 2). Next, we further stratified STH-infected subjects based on the number of STH species a subject was infected with at baseline. In subjects with multiple STH infections, albendazole significantly increased L/A ratio (1.25 (1.06–1.47), *P*=0.042), which derived from a significant reduction in adiponectin level (0.88 (0.80–0.97), *P*=0.015) and a non-significant increase in leptin level (1.10 (0.97–1.25), *P*=0.463) (Supplementary Figure 3). Using microscopy, a more pronounced reduction in adiponectin (0.90 (0.81–1.00), *P*=0.041) was observed in subjects infected with multiple STH species. The treatment effect on L/A ratio (1.15 (0.95–1.39), *P*=0.135) and leptin level (1.04 (0.90–1.20), *P*=0.677) in subjects infected with multiple species did not reach statistical significance (Supplementary Figure 4).

## Discussion

Our study is the first to report the effect of anthelmintic treatment on serum adipokine levels. In STH-infected subjects, treatment significantly increased L/A ratio, which has been reported to be associated with low-grade inflammation^16^ and IR.^16, 17^ The increased L/A ratio was derived by the significant reduction in adiponectin level, and to a lesser extent, a trend of increase in leptin level. As adiponectin induces the secretion of anti-inflammatory cytokines,^15^ while leptin increases Th1, suppresses Th2, and can act as a negative signal for the proliferation of human T regulatory cells,^20^ these changes may reverse the helminth-associated type 2 and regulatory immune responses, and presumably contribute to the development of IR. Indeed, adjustment for the increase in L/A ratio attenuated the treatment-associated increase in IR, observed in the main trial,^9^ even more than adjustment for increase in body mass index. This suggests that adipokines have a relatively more important role than the adiposity in the mediation of helminth-associated beneficial effect on IR.

Using PCR, a more sensitive method, able to detect non-clinically relevant STH infections, the treatment effects were less in magnitude, as it significantly reduced adiponectin level only, but to a lesser extent. In line with this, in subjects with multiple STH infections, associated with a higher infection intensity,^9^ treatment resulted in more pronounced effects, namely a significant reduction in adiponectin level, a trend for increase in leptin level, as well as a significant increase in L/A ratio. Except for the effect on adiponectin, these pronounced treatment effects were not observed when infection was assessed by microscopy, which might be due to the lower number of subjects who were found to be infected with multiple species, when using microscopy.

Despite having an ideal study design to study the causal relationship between helminth infections and adipokine levels, and to assess the contribution of adipokine levels to the increased IR after anthelmintic treatment, our study would have been more complete if we would have assessed food intake, appetite, and physical activity. In addition, measurements of other hormones that influence metabolism, such as ghrelin and cortisol, as well as analysis of AT biopsies and gut microbiome, could provide a more complete overview on how helminths may modulate human metabolism.

In conclusion, anthelmintic treatment in STH-infected subjects increases L/A ratio which may in small part contribute to the increased IR. Further studies will be needed to assess the effect of these changes in adipokine levels on the host metabolism and modulation of the host immune responses.

## Figures and Tables

**Table 1:** Study population

	*Placebo* N*=807*	*Albendazole* N*=750*
Age (in years, mean, s.d.)	41.9 (15.4)	42.6 (15.5)
Sex (female %, n/N)	62.0 (500/807)	59.9 (449/750)
Body mass index (kg m^−^^2^, mean, s.d.)	22.5 (4.0)	22.5 (4.0)
Leptin to adiponectin ratio (geomean (95% CI))	1.38 (1.25–1.53)	1.35 (1.21–1.51)
Leptin (ng ml^−1^) (geomean (95% CI))	7.1 (6.5–7.7)	6.7 (6.1–7.4)
Adiponectin (μg ml^−1^) (geomean (95% CI))	5.1 (4.9–5.4)	5.0 (4.7–5.3)
Resistin (ng ml^−1^) (geomean (95% CI))	15.6 (15.0–16.2)	15.7 (15.1–16.4)
Helminth-infected by microscopy (%, n/N)	43.5 (270/620)	40.5 (233/575)
Single species	28.2 (175/620)	26.4 (152/575)
Multiple species	15.3 (95/620)	14.1 (81/575)
Helminth-infected by PCR (%, n/N)	53.8 (392/729)	54.4 (368/676)
Single species	31.7 (231/729)	35.2 (238/676)
Multiple species	22.1 (161/729)	19.2 (130/676)

Abbreviation: CI, confidence interval.
