# Supplementary material for: Effect of anthelmintic treatment on leptin, adiponectin and leptin to adiponectin ratio: a randomized-controlled trial
Source: Nutr Diabetes. 2017 Oct 16;7(10):e289–. doi: 10.1038/nutd.2017.37 (PMC5678209; doi:10.1038/nutd.2017.37)
Supplement: Supplementary Information [file nutd201737x1.docx]

**SUPPLEMENTARY MATERIALS**

**Effect of anthelmintic treatment on leptin, adiponectin, and leptin to adiponectin ratio: a randomized controlled trial**

Dicky L. Tahapary^1,2,3,4^, Karin de Ruiter^2^, Ivonne Martin^5,6^, Eric A.T. Brienen^2^, Lisette van Lieshout^2^, Yenny Djuardi^3,7^, Clara C. Djimandjaja^3^, Jeanine J Houwing-Duistermaat^5,8^, Pradana Soewondo^1,4^, Erliyani Sartono^2^, Taniawati Supali^3,7#^, Johannes W.A. Smit^9,10#^, Maria Yazdanbakhsh^2#$^

1. Department of Internal Medicine, Division of Endocrinology, Faculty of Medicine Universitas Indonesia, Dr. Cipto Mangunkusumo National General Hospital, Jakarta, Indonesia.
2. Department of Parasitology, Leiden University Medical Center, Leiden, The Netherlands.
3. Nangapanda Community Research Cluster, The Indonesian Medical Education and Research Institute, Universitas Indonesia, Jakarta, Indonesia
4. Metabolic, Cardiovascular, and Aging Research Cluster, The Indonesian Medical Education and Research Institute, Universitas Indonesia, Jakarta, Indonesia
5. Department of Medical Statistics and Bioinformatics, Leiden University Medical Center, Leiden, The Netherlands.
6. Department of Mathematics, Parahyangan Catholic University, Bandung, Indonesia.
7. Department of Parasitology, Faculty of Medicine Universitas Indonesia, Jakarta, Indonesia.
8. Department of Statistics, University of Leeds, Leeds, United Kingdom
9. Department of Internal Medicine, Radboud University Medical Centre, Nijmegen, The Netherlands.
10. Department of Internal Medicine, Leiden University Medical Center, Leiden, The Netherlands.

^#^These authors have contributed equally, ^$^Corresponding author

**
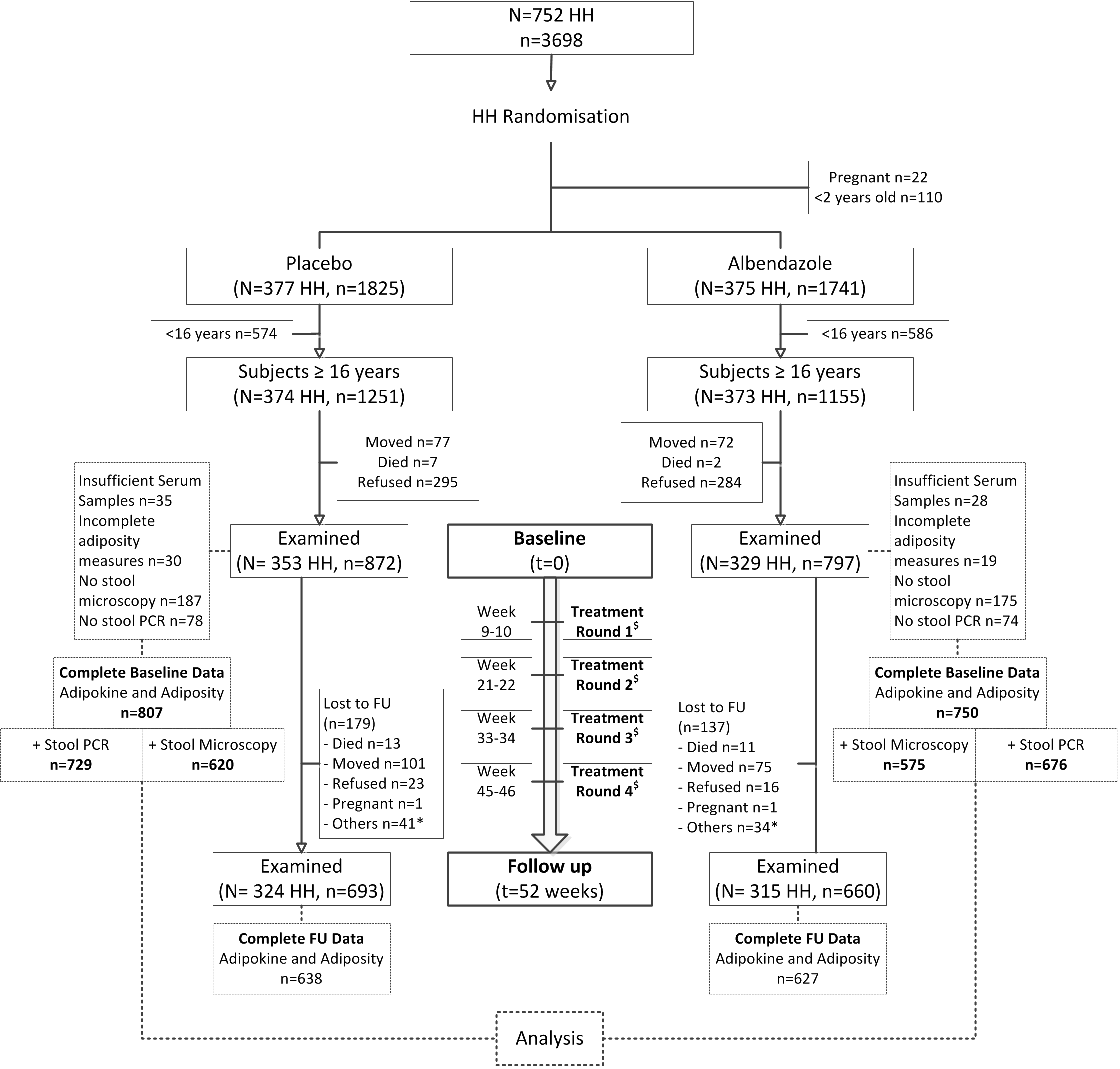
**

**Figure S1. Consort Diagram**

Baseline data (t=0) were collected during the first 8 weeks before the start of the drug administration. ^$^Single dose of albendazole or matching placebo was given for three consecutive days to all household members, except children below 2 years of age and pregnant women. *Other reasons of lost to follow-up were harvesting crops, working on funeral ceremonies, severely ill, hospitalized, nursing mother. Abbreviations: HH, Household; FU, Follow Up.

**Figure S2. Effect of anthelmintic treatment on adiponectin, leptin, resistin, and leptin to adiponectin ratio in soil-transmitted helminth (STH)-infected and uninfected subjects, as assessed by PCR.**

The effects of anthelmintic treatment on adiponectin (ADI), leptin (LEP), resistin (RES), and leptin to adiponectin ratio (L/A) are presented as proportion of changes between pre and post treatment in the albendazole group compared to the placebo group which is set to zero. The effects of treatment are presented for each group of subjects: (**A**) STH-uninfected and (**B**) STH-infected subjects, as assessed by PCR. Adiponectin, leptin, resistin, and L/A ratio were log-transformed for analysis. Analysis was performed on 1387 subjects, after excluding 14 subjects with diabetes. Treatment effect estimates were the regression coefficient (β) obtained from mixed models indicating changes in log (ADI or LEP or RES or L/A); the treatment effect factors (10^β^) are proportional instead of additive. Thus, treatment effect factors indicate the proportional change in each variable in comparison to the placebo group. *p<0.05.

**Figure S3. Effect of anthelmintic treatment on adiponectin, leptin, resistin, and leptin to adiponectin ratio stratified by number of helminth species a subject was infected with at baseline, as assessed by PCR.**

The effects of anthelmintic treatment on adiponectin (ADI), leptin (LEP), resistin (RES), and leptin to adiponectin ratio (L/A) are presented as proportion of changes between pre and post treatment in the albendazole group compared to the placebo group which is set to zero. The effects of treatment are presented for each group of STH-infected subjects with: (**A**) single STH species, (**B**) multiple STH species. Adiponectin, leptin, resistin, and L/A ratio were log-transformed for analysis. Analysis was performed on 1387 subjects, after excluding 14 subjects with diabetes. Treatment effect estimates were the regression coefficient (β) obtained from mixed models indicating changes in log (ADI or LEP or RES or L/A); the treatment effect factors (10^β^) are proportional instead of additive. Thus, treatment effect factors indicate the proportional change in each variable in comparison to the placebo group. *p<0.05.

**Figure S4. Effect of anthelmintic treatment on adiponectin, leptin, resistin, and leptin to adiponectin ratio stratified by number of helminth species a subject was infected with at baseline, as assessed by microscopy.**

The effects of anthelmintic treatment on adiponectin (ADI), leptin (LEP), resistin (RES), and leptin to adiponectin ratio (L/A) are presented as proportion of changes between pre and post treatment in the albendazole group compared to the placebo group which is set to zero. The effects of treatment are presented for each group of STH-infected subjects with: (**A**) single STH species, (**B**) multiple STH species. Adiponectin, leptin, resistin, and leptin to adiponectin ratio were log-transformed for analysis. Analysis was performed on 1183 subjects, after excluding 12 subjects with diabetes. Treatment effect estimates were the regression coefficient (β) obtained from mixed models indicating changes in log (ADI or LEP or RES or L/A); the treatment effect factors (10^β^) are proportional instead of additive. Thus, treatment effect factors indicate the proportional change in each variable in comparison to the placebo group. *p<0.05

**Table S1. Effect of Anthelmintic Treatment on Soil-transmitted Helminth Prevalence**

| **Method** | **Placebo** | | **Albendazole** | | **p-value*** |
| --- | --- | --- | --- | --- | --- |
|  | **Baseline** | **Follow-up** | **Baseline** | **Follow-up** |  |
| Microscopy (%, n/N) | 43.5% (270/620) | 26.8% (166/497) | 40.5% (233/575) | 5.2% (24/466) | <0.0001 |
| PCR (%, n/N) | 53.8% (392/729) | 45.0% (250/555) | 54.4% (368/676) | 10.4% (55/529) | <0.0001 |

*Analyzed using logistic model (lme4 package R software) with random household effects and random subject effects

**Table S2. Pathway analysis on the role of leptin to adiponectin ratio in the increased insulin resistance after anthelmintic treatment**

|  | **Crude** | **L/A Ratio** | **BMI** | **L/A Ratio + BMI** |
| --- | --- | --- | --- | --- |
| HOMA-IR* | 1.07 (1.01 – 1.14)  p=0.023 | 1.05 (0.99 – 1.11)  p=0.075 | 1.06 (1.00 – 1.12)  p=0.048 | 1.05 (0.99 – 1.11)  p=0.075 |

*Analyses were performed using linear mixed model in unadjusted model (crude) and adjusted for leptin to adiponectin (L/A) ratio, BMI, or both.

**Contribution statement**

D.L.T. is a medical doctor in charge of the field study, involved in setting up the study, supervising gathering of data, treatment, clinical care, follow up of the study population, analyzed the data and wrote the manuscript. K.R. is a medical biologist in charge of the field study, involved in setting up the laboratory in the study area, performing the immunological analysis, supervising the data cleaning, and the follow up of the study population. I.M. is a mathematician who is developing methods to analyze the complex data generated during the lifetime of the project and was involved in the randomization and data analysis. L.v.L. is a parasitologist who was involved in the performance and analysis of diagnostic assays for the detection of helminths in stool samples. E.A.T.B. is a technician who develop, optimized and performed multiplex real time PCR for detection of helminth infections. P.S. is an endocrinologist who advised on the metabolic aspects of the study. Y.D. is a medical doctor who was involved in coordinating the field study and advised on the immunological and parasitological aspects of the study. C.C.D. is a nurse who was involved in the field study, especially gathering of data, supervising anthelmintic treatment, and follow up the study population. J.J.H. is a biostatistician who developed the study, and was involved in supervising sample size calculation, randomization and statistical analysis. E.S. is an immunoparasitologist who was involved in coordinating the study and advising on parasitological and immunological aspects of the study and supervised the writing of the manuscript. T.S. is a parasitologist who developed the study and is the Indonesian coordinator of the SUGARSPIN program. J.W.A.S. is an endocrinologist who developed the study, supervised the writing of the manuscript, and is the Dutch coordinator of the SUGARSPIN program. M.Y. is an immunologist who developed the study, supervised the writing of the manuscript and is the scientific coordinator of the SUGARSPIN program. All authors read and approved the final manuscript.
